# Sexual harassment at work: Targets’ perspectives on prevention and response

**DOI:** 10.1371/journal.pone.0352783

**Published:** 2026-07-10

**Authors:** Christianne Corbett, Meghan O. Warner, Sarah J. Harsey, Jennifer J. Freyd

**Affiliations:** 1 Department of Sociology and Criminology, University of Arkansas, Fayetteville, Arkansas, United States of America; 2 Stanford Introductory Studies, Stanford University, Stanford, California, United States of America; 3 School of Psychological Science, Oregon State University – Cascades, Bend, Oregon, United States of America; 4 University of Washington and Center for Institutional Courage, Seattle, Washington, United States of America; Higher Education Partnership / Erasmus University Rotterdam, ETHIOPIA

## Abstract

Harassment, including sexual harassment, in the workplace poses a significant threat to workers’ wellbeing and contributes to social inequities. Organizations play a pivotal role in both enabling and preventing workplace harassment. We use a grounded theory approach to understand how workers experience workplace sexual harassment. Drawing on semi-structured interviews with thirty U.S. workers, most from the service industry in the San Francisco Bay Area, who have experienced sexual harassment at work, we describe organizational practices that workers believe effectively prevent and respond to harassment. Findings demonstrate that workplaces may better support workers by (1) setting clear expectations that harassment will not be tolerated and (2) responding effectively to incidents of harassment by establishing an independent entity to whom victims can report incidents, providing verbal support, and taking appropriate actions to support and protect victims.

## Introduction

Social scientists argue that harassment – including sexual harassment – is both a cause and a consequence of social inequality [1,2]. While much research examines this issue from the lens of gender inequality, harassment also reproduces inequality by race and ethnicity, class, ability, age, sexual identity, and citizenship status. Those who are marginalized along these dimensions are more likely to experience harassment [3]. People who experience workplace harassment often face negative social, emotional, mental, physical, and financial outcomes [4,5]. In short, harassment maintains and reproduces inequality in the workplace and beyond.

In this paper, we ask: what do victimized people working in the United States believe are effective organizational practices to address the problem of sexual harassment at work? Based on semi-structured interviews with 30 workers, most from the service industry in the San Francisco Bay area, who experienced sexual harassment at work, we identify several organizational practices related to both prevention of and response to sexual harassment that may help workers feel supported at work. With respect to prevention, participant responses suggest that workers feel more supported and safer when organizations make their expectations of worker behavior clear before any incident of harassment occurs. In terms of response, findings suggest that workers feel more supported and safer when organizations put in place an independent actor to whom workers can report incidents, provide verbal acknowledgement and support when workers report experiencing harassment at work, and follow up with appropriate actions. Organizations that fail to formalize these steps may exacerbate the initial harm perpetrated on workers who experience harassment. Moreover, organizations that incorporate these practices may mitigate such harm by both reducing the likelihood that workers will experience sexual harassment at work and supporting those who do. Although only one piece of the puzzle, understanding victims’ perspectives is a crucial element to identifying organizational practices that mitigate the harms of sexual harassment.

### Sexual harassment at work

Unwanted attention at work comes in multiple forms including bullying and harassment. Anyone can experience unwanted attention at work, but it is more common among individuals who are deemed lower status by virtue of some aspect of their identity such as gender, race, or sexual orientation [6,7]. The literature on unwanted attention focuses primarily on unwanted gender-based or sexual attention (i.e., sexual harassment), a pervasive social problem that adversely impacts a significant number of people. A key finding from this literature is that sexual harassment is typically rooted in power and gender dynamics rather than sexual attraction [8]. Drawing on this finding, Berdahl [9] argues that sexual harassment should be viewed “as behavior that derogates, demeans, or humiliates an individual based on that individual’s sex.” (p.644). Following the tripartite model of sexual harassment [10,11] throughout this paper, we use the term “sexual harassment” to refer to any one of three interrelated but distinct phenomena: sexual coercion (quid pro quo scenarios such as threats of retaliation for not complying with requests for sex), unwanted sexual attention (sexual behaviors that are unwelcome and unreciprocated), or gender-based harassment (acts of aggression, intimidation or hostility based on gender, gender identity, or sexual orientation often aimed at reinforcing heteronormative gender roles) [12]. Nationally representative surveys find high rates of sexual harassment. In a recent survey, 38% of women and 14% of men report experiencing sexual harassment at work [13]. Other estimates suggest about 50% of women experience sexual harassment [14]. Workplace sexual harassment continues to plague workers despite decades of advocacy and legal reform.

Workplace sexual harassment in the U.S. violates Title VII of the Civil Rights Act of 1964.The U.S. Equal Employment Opportunity Commission (EEOC) recognizes two types of sexual harassment: (1) *quid pro quo*, when “submission to or rejection of such conduct by an individual is used as the basis for employment decisions affecting such individual” and (2) *hostile environment*, or behavior that is “sufficiently severe or pervasive to alter the conditions of [the victim’s] employment and create an abusive working environment” [15]. The EEOC notes that behavior does not need to be sexual in nature to constitute a violation of Title VII – it can include offensive remarks based on a person’s sex, which Leskinen et al. [16] argue is the most common form of workplace gender-based harassment in many fields. Importantly, we also note that workplace sexual harassment does not constitute a legal violation in a substantial minority of other countries; for instance, workplace sexual harassment is legal in 22% of high-income countries and 34% of low-income countries [17]. The framing of this study and the interpretation of its findings therefore occur in a U.S.-specific context in which sexual harassment in the workplace is illegal.

### Consequences of workplace harassment

Workplace harassment research often focuses on consequences for the victimized person. (In the title of this paper, we use “target” to refer to the individual experiencing harassment. Throughout, we use “victim,” “victimized person,” “survivor,” or “target” interchangeably as all terms are used in the literature to refer to an individual experiencing harassment. See Warner [18] for more on labels for people who have experienced sexual violence.) Those who are harassed can experience short-term and long-term distress [19,20]. Negative emotional consequences can include anger, fear, sadness and depression, and humiliation and distrust [21]. Trauma symptoms and psychosomatic symptoms have also been found to be associated with experiencing harassment [22–24]. The severity and duration of these outcomes can vary depending on characteristics of the harassment. For instance, individuals who are harassed by perpetrators with greater organizational power report greater stress and, consequently, worse psychological wellbeing [25]; research also suggests that negative outcomes increase as the severity of harassment increases [16]. Institutional status of all parties also influences outcomes, and this status is impacted by common markers of difference such as race, class, and gender [7].

Scholars have also documented consequences specific to workplace productivity and commitment. People who have experienced harassment in their line of work report decreased job satisfaction and performance [21,26–28]. These outcomes are partially explained by victims’ decreased attachment and commitment to the workplace [24], which can result in showing up late for work, decreased productivity, and seeking employment elsewhere. Longer-term consequences of workplace harassment include financial distress and career interruptions [5]. Beyond individual-level outcomes, harassment is also tied to increased team conflict and decreased team financial performance [29].

### Organizational factors

Organizational factors can impact the prevalence of harassment. Sexual harassment has been found to be more common in hierarchical workplaces with strong power differences between organizational levels [30] and in workplaces dominated by men or masculine norms [31]. Meta-analyses reveal that the strongest predictor of workplace sexual harassment is the organizational climate, or its tolerance for sexual harassing behaviors [24,32]. Sexual harassment is more likely to occur in workplace climates that support gender inequity [33], offer inadequate resources or education for workers, or fail to effectively implement sexual harassment policies [21,34]. Certain industries, such as service and hospitality, are particularly likely to foster organizational climates that both enable sexual harassment and fail to address it, due in part to structural power imbalances and “customer is king” ideologies that often prioritize guest satisfaction over worker safety [35,36] Illustrating this, findings from experiments indicate that employers perceive sexual harassment less negatively when it is committed by customers [37]. On the other hand, evidence-based sexual harassment education encouraged by the organization may reduce prevalence of sexual harassment and increase reporting rates [38,39, but see 40]. Worker perception of sexual harassment policies and procedures also matters, contributing to both the prevalence of harassment and its impacts [34]. When workers perceive that reporting sexual harassment is risky or that reports will not be taken seriously, they also believe their workplace is more likely to foster a climate supporting sexual harassment [41]. In a review, Pina and Gannon [21] argue that unsupportive workplaces lead to both fewer reports and a greater prevalence of sexual harassment.

Additionally, workplace leadership can shape the organizational climate related to harassment. Hart, Crossley, and Correll [42] experimentally tested the effect of leadership messaging on individuals’ perceptions about the importance of sexual harassment as a problem. Individuals who read a leadership statement framing sexual harassment as a serious issue were more likely to consider it to be an important community problem compared to those who were exposed to a leadership statement that downplayed its severity.

Organizational climate factors are closely tied to reporting rates. People do not report harassment for a variety of reasons, including under-labeling of the harassment [43,44]. Workers may also be less likely to report harassment when policies are vague or nonexistent [45] because they think their experiences do not warrant reporting, they do not trust that the process will be fair and impartial, and/or they think reporting harassment will not yield any positive results, including stopping the behavior [45,46].

Those who do report harassment often experience negative personal and professional outcomes [47]. People often fear negative consequences from reporting harassment, including retaliation and reputational damage, and research findings underscore the legitimacy of these fears [46,47]. For example, in an experiment, women who indicated they had experienced sexual harassment were less likely to be recommended for promotion [48], demonstrating how workplaces often fail to appropriately respond to sexual harassment.

Negative and ineffective organizational responses to maltreatment – such as sexual harassment in the workplace – can make outcomes associated with maltreatment, including mental and physical health outcomes, worse [49–51]. This is the central tenet of the concept of institutional betrayal, which describes how institutions on which individuals depend (e.g., universities, workplaces, or religious institutions) cause harm when they fail to either prevent or respond supportively to maltreatment experienced within their organizations [52]. Institutional betrayal research specific to workplace harassment is scarce. However, early research suggests institutional betrayal in response to workplace harassment is associated with poor job satisfaction, decreased organizational commitment, increased work withdrawal, decreased intention to remain with the organization, and poor mental and physical health outcomes [53]. Research on institutional betrayal in the military, an especially all-encompassing work environment, provides some initial evidence that inadequate organizational responses to worker claims of sexual maltreatment may have disastrous effects. Institutional betrayal following military sexual trauma is associated with more severe depression and specific posttraumatic stress disorder symptom clusters [54], and perceptions of institutional betrayal predict suicidal self-directed violence among veterans exposed to military sexual trauma [55].

On the other hand, certain workplace responses have been found to ameliorate harmful outcomes following harassment [56]. Smidt, Adams-Clark, and Freyd [53] find that organizational responses that demonstrate “institutional courage,” a set of behaviors focused on protecting and caring for the individuals who depend upon the institution, were correlated with greater job satisfaction, organizational commitment, and trust in management among workers. In short, this research suggests organizational practices are consequential for the harm that results from harassment. Building on this literature, as well as the literature on the causes and consequences of workplace sexual harassment, this study explores the lived experiences of individuals who have experienced sexual harassment at work. We seek rare cases where people who have experienced sexual harassment at work also experienced support that felt like institutional courage to shed light on organizational practices that can prevent workplace harassment and effectively respond to it when it happens.

### Data and methods

Participants were 30 people recruited through online posts on the networking websites Craigslist and Nextdoor between October 7, 2019 and July 15, 2020. All respondents live in the U.S., all but one in the San Francisco Bay Area of California. Our ad invited individuals who had experienced “unwanted sexual attention or behavior that made you uncomfortable” at work to participate in a confidential 60–90 minute interview. Below the invitation was a short paragraph saying we are a team of “researchers interested in learning more about your experience.” The ad offered a $50 Visa gift card as compensation and included a contact email address. The initial recruitment message sought participants who worked in the restaurant, hotel, or hospitality industries as research has found that harassment is widespread in these industries [35]. We later removed that criterion to attract more participants and to better understand experiences and commonalities across a variety of industries.

Participants were 19–56 years old, with nearly half (14 participants) in their 30s. Of the 30 participants, 18 were women, 10 were men, and two were gender non-conforming or gender-fluid. Eighteen had at least a bachelor’s degree, and the rest had an associate’s degree or had attended some college. Nine participants identified as Asian or Asian Pacific Islander, nine as White, six participants identified as Black or African American, five as Latinx or Hispanic, and one as biracial. They worked in a variety of industries, most of which are in the service sector, including food service, healthcare, technology, and retail (See Table 1). Most of the participants were at low-mid levels in their organizations at the time of the harassment that they described to us.

**Table 1:** Respondent Characteristics & Employment Industry at Time of Interview.

Gender		Race		Age		Industry	
Women	18	Asian	9	<20	1	Service	
Men	10	White	9	20-29	5	Restaurant	7
Non-binary	2	Black	6	30-39	14	Healthcare	4
		Latinx	5	40-49	7	Information Technology	3
		Biracial	1	50-56	3	Retail	2
						Hotel	1
						Childcare	1
						Call center	1
						Office staff	1
						Professional & Business	
						Management	2
						Engineering	2
						Energy	1
						Marketing	1
						Political campaign	1
						Non-profit	
						Non-profit worker	2
						Art	
						Artist	1

All 30 participants experienced harassment at work, which ranged from verbal behaviors such as gender-based or sexual comments to physical violations, including rape. Only 17 participants reported an incident of workplace sexual harassment to a supervisor, a Human Resources (HR) department, or another colleague at work. While these reports encompassed both formal reporting procedures and informal disclosures to colleagues, most (88%) participants who reported sexual harassment did so to those with a legal responsibility to take action (i.e., a supervisor or other workplace representative). In the US, employers are legally obligated to investigate claims of sexual harassment and, when appropriate, respond with corrective actions. The remaining 13 participants did not tell any authority or anyone with whom they worked about the sexual harassment they experienced.

### Ethics approval and consent to participate

All study procedures were approved by Stanford University’s Research Compliance Office (Institutional Review Board). Our procedures were designed to protect confidentiality to minimize risks of workplace retaliation against participants. To avoid written documentation linking participants’ real names to participation in the study, informed consent was obtained verbally. Immediately prior to each interview, participants were provided with an information sheet about the study either printed (if interviewed in-person) or via email (if interviewed virtually) and asked to read the sheet in full. After each participant read the information sheet, the interviewer verbally asked if they agreed to participate in the study, and all participants verbally consented to participate in the study. Participants were assured identifying information would remain confidential, and only anonymized portions of their transcripts would be published. The interviewer then asked if they agreed to have the interview recorded, and again, all participants agreed. At the end of each interview, participants were asked demographic questions regarding their age, race/ethnicity, gender, level of education, and sexual orientation. Categorization of participants into various identity groups was done by the participants’ self-description. In addition, participants were provided a list of resources relating to workplace sexual harassment at the end of the interview. Some of the resources, such as the Silicon Valley YWCA, offered counseling services. No participants dropped out over the course of the research.

### Materials and procedures

Interviews were conducted either in person or virtually via video-conferencing (mostly on Zoom), with, to the best of our knowledge, only the interviewer and interviewee present. We detected no differences between in-person and zoom interviews. Interviews were conducted by one of three Sociology PhD candidates, all of whom are White women. Interviews typically lasted about an hour, and we asked participants about their experience(s) with unwanted attention, how it impacted them, and how they addressed the issue. We sought to understand company culture, as well as specifics around company policies and procedures, if applicable. We include the interview protocol as supporting information (See S1 File. Interview Protocol). Because these interviews were semi-structured, we were able to adapt questions to apply to each individual’s specific situation. We then wrote memos about each interview (usually approximately one page long), which allowed us to record aspects of the interview missed through audio.

We transcribed the interviews using a transcription service (Keltran). Using a grounded theory approach, the second author coded the interview transcripts with NVivo, a software coding program,  marking key aspects of each transcript with descriptive codes that emerged from the interviews, guided by our research question and analytical memos [57]. We include our coding guide in the supporting information (See S2 File. Interview Codebook). The first author then analyzed the coded transcripts, allowing themes to emerge from the data. We developed our initial interview questions based on our knowledge of prior research on sexual harassment as well as our conceptualization of institutional courage as a buffer to workplace harm from sexual harassment and institutional betrayal. The project is thus based in the idea that organizations can take actions to prevent and mitigate some of the harm of gender-based and sexual harassment, making those experiences both less likely to occur in the first place and more manageable for workers when they do occur. In this paper, we focus on understanding how participants described and made sense of their experiences and their work environments to build a theory of institutional courage grounded in worker experiences [58].

## Results

As they talked about their workplace experiences, participants described organizational practices they perceived as effective in 1) preventing sexual harassment at work and 2) responding to incidents of harassment when they happened. Although a strict distinction between prevention and response is artificial since aspects of effective responding will likely contribute to prevention of future harassment, we organize our results section according to these two aspects because both preventive and responsive practices are important for effectively answering our research question.

### Prevention

First, because most of our participants had worked in multiple organizations and had rarely experienced sexual harassment in all of their workplaces, they were able to talk about organizational practices that seemed to reduce the likelihood of experiencing sexual harassment at work and to support them proactively.

#### Set clear expectations.

One such practice that emerged from our data is clear messaging from organizational leaders – before any incident occurs – that sexual harassment of co-workers is not acceptable. Participants described how proactive discussions of sexual harassment by leadership communicates that leaders are aware that it can happen, do not condone it, and that there will be negative consequences for workers who violate organizational expectations about it.

For example, Mai, a 34-year old Asian woman, who had experienced harassment at several previous jobs in lawncare, childcare, and at a for-profit higher-education company, described the lack of sexual harassment at her current workplace, a solar panel company:

We don’t have no issues…My company, the GM’s and the Service Managers and the Production Managers, and just, you know, the President, when we are on the call, they are always saying ‘Okay, you know, we’re here to work hard, we can play hard, but play with boundaries’… So they always, you know, are talking about it… I feel like it’s on their radar…. he’s like ‘You guys have to behave well, because even though it’s not work, it’s still [a] work event…. do not do any sexual harassment things because I do not want to let you go for something that you have fun for a split second that will cost you your career’. (Mai)

Mai appreciated frequent communication from leadership regarding workplace behavioral expectations. In this example, management downplayed the seriousness of harassment – describing it as “fun” for the perpetrator – which might contribute to an environment where victims decide not to report incidents of harassment. At the same time, she believed such language might reach potential perpetrators and encourage them to behave more respectfully. Even though she recalls flippant language regarding sexual harassment, Mai still felt supported by the reminder that such behavior was inappropriate. Mai’s example demonstrates that language around preventing workplace sexual harassment need not be overly formal to be effective for some workers.

Gabrielle, a 35-year-old Black woman who had experienced unwanted sexual attention at previous jobs, described a similar culture of open discussion of harassment by leadership on a political campaign where she had been employed prior to the interview:

It is something that is always spoken about. There is a lot of transparency around it… people are just really honest about sexual harassment, all their directors are real open about anonymous information…they wanted to make sure that they could address people who felt uncomfortable or bullied or so many different things, whether it be your sex, your race, your class, your performance or your experience. (Gabrielle)

Gabrielle felt supported by her organizational leadership’s frequent discussion of sexual harassment. Leadership’s “transparency” included providing opportunities to anonymously report experiences of unwanted attention, including forms of unwanted attention other than sexual harassment. Gabrielle and others felt supported by frequent and open conversation about sexual harassment at work.

Organizations may attempt to make clear their behavioral expectations and resource options through education about sexual harassment. However, workplace harassment laws in the US have incentivized employers to adopt anti-harassment trainings primarily as a means to reduce legal liability, which may not necessarily translate to effective reductions in harassment [59]. Thirteen participants (43%) expressed frustration (using terms like “boring” or “stupid”) over anti-harassment training they saw as an attempt by the organization to “check the box” of following the law. On the other hand, eight respondents (27%) described education about sexual harassment that they saw as effective [38]. Stephanie, a 41-year old Asian woman, discussed effective education about harassment. She had experienced routine harassment at a previous job in television. In lieu of any effective education about harassment or enforcement of anti-harassment rules, Stephanie found herself performing the additional labor of managing her appearance by wearing “frumpy” clothes as a way to prevent any unwanted attention, which increased her anxiety and stress on the job. At a new workplace, she described being surprised and pleased by the education about harassment:

When I signed up to be an extra in [a state film project] they did send me an online sexual harassment training and it was probably 120 slides and I was shocked because I mean I just signed up to be an extra on movies and then…. they sent me this long training about ‘you can come to us with any issues, harassment is wrong’ and, yeah, I was shocked that they had this whole presentation….’You don’t deserve to be harassed’ and everything. I’ve never seen any kind of messaging like that before. So that was different… I was just surprised that anyone would care. Because usually harassment is normal. Because women deal with it every day and they just have to brush it off. It’s just emotional baggage they have to leave behind. (Stephanie)

Did you feel like it might make harassment less likely in that environment? (Interviewer)

I think so, yeah because they were trying to get across the point that you have rights that you don’t need to be harassed and I think a lot of women don’t hear that because a lot of women just [think] ‘Even if I complain no one will care’ which usually people don’t do. People usually don’t care because everyone else is being harassed too. Who has time to deal with – you would probably be sitting there all day long with all the harassment calls or whatever. (Stephanie)

At her newest job, she felt supported by messaging that she did not “deserve” unwanted attention at work, and she had rights if someone treated her inappropriately. She connected this education to her previous experiences being harassed at workplaces that did not provide messaging about sexual violence. She was pleased her temporary workplace offered reporting resources instead of dismissing this responsibility as one that overwhelmed the organization.

Focusing on prevention through open and transparent communication and education about sexual harassment takes the onus for addressing acts of harassment at work off the shoulders of individual workers and puts it on the organization. Our data suggest that clear messages from leaders about expected behavior is one element of creating a work culture where all workers, and especially women who are disproportionally affected by sexual harassment, are respected and listened to. By specifying the kinds of behaviors that are acceptable and those that are not, organizational leaders can make clear the boundaries that all workers are expected to respect. Because workers may have different ideas of where those boundaries are, participants believed clear organizational messaging might establish appropriate norms and reduce workers’ negative experiences at work. While scholars disagree about the extent to which miscommunication leads to sexual harassment [60], organizationally stated boundaries may reduce sexual harassment by helping create norms and assuring workers that all forms of sexual harassment are unacceptable. Prevention efforts like these may reduce incidents of sexual harassment at work, but they may not fully eradicate them. In some cases, they may even create new risks for workers. For instance, qualitative work in the film and television industry suggests that workers who are encouraged to disclose experiences of workplace sexual harassment face retaliation after doing so [40]. Findings like this indicate that organizational messaging may be insufficient without appropriate organizational responses. In the next section, we describe organizational responses that felt supportive to workers who experienced sexual harassment.

### Response

As reported above, although all the participants in our study experienced sexual harassment at work, just under half (13/30, or 43%) of respondents never reported the incident(s) to anyone at their organizations. As we might expect with a qualitative studying seeking people who want to discuss unwanted attention at work, the 57% of our sample that reported harassment to someone in their organization is higher than the approximately 12.5% of women and 5% of men in the general population who filed a formal complaint about their experience(s) with sexual harassment [13]. This relatively high reporting rate allowed us to analyze participants’ views about effective responses to sexual harassment based on their reporting experiences. Our analysis of the interview data revealed three organizational practices that participants found supportive when they experienced sexual harassment at work: First, having an independent entity for reporting helped workers feel safer reporting incidents of harassment compared to reporting to an organizational colleague. Next, rather than letting claims of sexual harassment “slip under the rug,” as one participant said, participants indicated they felt supported when organizational representatives provided verbal support. Finally, participants said they felt supported when organizational representatives followed up with appropriate actions to protect the victimized person. All three of these practices are important, and one or two without the other(s) might undermine any potential benefit from implementing only a subset of them. For example, one participant described how verbal support without follow-up actions from her employer felt condescending.

#### Establish independent entity for reporting.

Five participants (17%) described the importance of having someone trustworthy outside their chain of command to whom to report sexual harassment incidents. External reporting mechanisms are typically structurally separated from workers’ immediate workplace hierarchies, and may include unions, ombudsmen, or third-party consulting firms. In some cases, even internal HR personnel or workplace representatives may be perceived as a viable reporting option as long as they operate independently from the chain of command. Generally, however, participants talked about how they felt unable to report incidents of sexual harassment to anyone in their organization because there was no safe person to whom to report them. John, a 34-year-old Asian man, described feeling reluctant to report the harassment he experienced due to the lack of an external or independent entity.

I just kept my mouth shut because if I said anything, I was like, “Okay, it could help in the sense of it may get rid of this guy, but the other chance is that I... the bigger chance is that I definitely will get put on some sort of leave or something like that, which I can’t afford. I’m living paycheck to paycheck, so I can’t be out of work for a week.” (John)

John highlights the difficult and precarious situation many workers find themselves in when they fear retaliation and when the organization lacks an independent body to whom to report incidents of harassment. John describes feeling like there was a tradeoff between safety on the job and security of income. Like other participants, John describes how he did not report the harassment because he could not risk losing his income.

Participants described receiving unwanted sexual attention from the owner of their place of employment or his/her close colleagues. This pattern was especially common among workers in small, owner-run businesses. In these and other cases, respondents often referred to a perceived positive relationship between the perpetrator and the investigative personnel as a reason they did not report. For example, Matthew, a 50-year-old man who identified his racial or ethnic background as Chinese, said:

That department should be complete[ly] independent on its own. They shouldn’t report to anyone, because it can be the chief. The chief can harass other people, then who can you report to? And…those need to be confidential. The chief doesn’t need to know about those. (Matthew)

Matthew acknowledged the role workplace hierarchies can play in protecting people high up the hierarchy who harass others [30]. Like others, Matthew suggests a “completely independent” entity to avoid the power dynamics inherent in a hierarchical workplace model.

On the other hand, Gabrielle, the 35-year-old Black woman mentioned earlier, described feeling safe working for a political campaign that had multiple choices for workers to report sexual harassment:

[You] could report to the HR manager or director either and for each of these, they said you could either do it for the state you are in or headquarters, depending on the person, one could feel more comfortable than the other. And then, they gave us the numbers for the union and our consultant firm that came in to do mediation….They also made themselves available should somebody want to report something back and have it be completely outside of the organization and, again, is confidential. (Gabrielle)

In contrast to John and Matthew, Gabrielle describes how her employer communicated to workers that they could report incidents of harassment to various entities. By allowing workers to choose an individual to whom they felt comfortable reporting harassment from among several options, Gabrielle’s employer reduced the likelihood that workers would not report out of fears of retaliation.

#### Provide verbal acknowledgement and support.

In addition to highlighting organizational characteristics that either discouraged reporting or made reporting feel more accessible should sexual harassment occur, participants also detailed helpful responses they experienced following disclosures of sexual harassment to their employers. Participants felt supported when organizational representatives listened, acknowledged and validated their experiences, took their claims seriously, and expressed how the harassment was unacceptable. Ruby, a 31-year-old Latina temporary child-care worker, discussed how a client had harassed her in his home when she was caring for his child. Ruby described feeling supported by her employer’s verbal response when she reported the incident via text:

They were like ‘oh, my gosh.’ So they directly called me right after because the app is just strictly texting I guess. So they called me and said ‘explain what’s going on.’ And now I was like ‘okay, I’m sorry’, I had to calm down. They’re like ‘No, take your time, you know, it’s, you know, understandable.’ They’re being so, you know, understand[ing], you know, for the whole situation. So I explained to them like what happened…They said they were going to handle this […] like ‘we’ve got to take him out of the database. We’re going to talk to him about this. This is not okay. This is dangerous to our providers, you know. We don’t want to allow this in the company.’ Like, you know, ‘we care about our providers and their safety and, of course, the children….this person is not going to be allowed to even use any babysitting apps because we don’t know who he is or what background he has’ or whatever. (Ruby)

Ruby immediately reported experiencing workplace harassment, and a representative from her employing organization responded quickly and empathetically. She said the organization was “understanding” and reiterated that the harassing behavior was unacceptable. They quickly acted by removing the offender from their database. Ruby described feeling supported by this organization.

Similarly, Alexis, a 21-year old White coffee shop barista, described how she felt supported by her supervisor, Alexandra, when she told Alexandra about a customer who was harassing her. Alexis said:

I know she will do the right thing, so I just let her know it was happening and she was like, ‘okay, yeah, that’s a problem. We know about it. Thanks for letting me know. I’ll do everything I can to stop it.’ And then that conversation ultimately went up to the store leader and he kind of banned him [the harassing customer] for a while. And since that, I kind of let all the new girls know, especially since they are like teenagers or underage, I let them know that if he does that or anyone else does that, let Alexandra know, because I don’t want them feeling the way that I did, like stuck in that situation….[Alexandra] listens to me. She looks me in the eye….she asks me questions about it too so she seems like interested in it, but she’s not like, ‘oh, tell me more.’ It’s just…to get the facts and make sure that like it is actually happening and that it is a problem for me. (Alexis)

Like Ruby, Alexis felt supported by her employer when she reported experiencing harassment from someone outside of the organization. Her boss listened to her, asked follow-up questions, and took action to prevent the harassment from occurring again.

Participants were also asked to describe what they thought would be an appropriate company response to reported workplace harassment. Basing their responses in their experiences of reporting, several participants mentioned that the organization should apologize, explaining either that they would have liked an apology or that they appreciated when their employer or an organizational member apologized. Approximately half of participants who reported incidents to someone at their organization received some form of apology from an organizational representative. Although eight participants said they did not receive an apology from anyone in their organization, two reported receiving apologies directly from the person who had harassed them or made them feel uncomfortable, five received apologies from management, and two received an apology from HR. Katie, a musician, described how she felt “very acknowledged” after receiving an apology from a colleague who made an offensive comment to her, and that as a result “the interaction ended up being far less uncomfortable.” Employers likely hesitate to make apologies at least in part because they fear the liability that goes along with such an admission of wrongdoing. Nonetheless, our findings speak to the power apologies may possess for redressing the harm of harassment. Maria, a 29-year old Mexican woman, described the kind of apology she wished she would have received from her manager:

…that would be [my manager] being like ‘Hey, I’m really sorry. I can see now from your perspective how that’s really inappropriate and even me talking about women the way I do is really inappropriate’ and, yeah, like ‘I’m going to change. I’m not going to do that anymore. That’s really – that’s not cool, it’s not funny and this is a business and we just – we don’t need to do that.’ (Maria)

Maria wanted her manager, who had harassed her, to take responsibility for his behavior and demonstrate accountability by promising not to do it again.

In contrast, participants described dissatisfaction, disbelief, and sometimes disgust when they did not receive acknowledgement or validation from their employers. Amber, a 34-year-old White paramedic who works on an ambulance, described a situation in which her employer did not acknowledge a report made by her colleague, Anna, to her supervisor that she (Anna) had been raped by a co-worker. The supervisor did nothing until Anna brought it up with the CEO who then fired the co-worker who Anna reported had raped her. The management made no statement about the co-worker’s behavior to the rest of the organization, and many of the men with whom Amber and Anna worked talked badly about Anna, retaliating against her for reporting their co-worker. In this way, in addition to being raped by a colleague, Anna was further harmed by being the subject of negative gossip at work. Amber said:

There was no statement put out about how a member of management had acted inappropriately and maybe the company should apologize for not dealing with it. And maybe this shouldn’t happen in the future. We’ve heard nothing from management. And once you [asked about employer response], I was like, ‘wow, that is crazy that they didn’t even bother drafting it, like just shoved it under the rug.’ (Amber)

Although Anna’s organization eventually removed the perpetrator, her colleague, Amber, felt unsafe because the situation had never been explained to other employees. Amber’s assessment demonstrates the importance of both timely communication and transparency regarding workplace sexual harassment.

#### Follow up with appropriate actions.

Finally, our analysis of participant interviews suggests that organizations’ follow-up actions are critical to supporting workers who report sexual harassment at work, even if the follow-up happens well after the incident took place. Participants described how they felt supported after reporting harassment when their employer checked in with them and offered to provide any services they might need. For example, Rose, a 38-year-old Filipino nurse, described experiencing a situation at work around twenty years ago in which a group of more senior nurses physically threatened and bullied her, making her work without breaks and stay late. Although not sexual harassment, such unwanted attention involving bullying and threatening behavior is another type of legally prohibited harassment. Rose described how HR reached out to her, much later, after she reported the unwanted behavior to her manager, saying:

’Hey, are you okay?’ They offered me services and I’m like, ‘You know, this happened so long ago. I’m good.’ Um, and then they just told me what services were available to me. And then like ‘if it happens again, this is what’ll happen or what can happen next time’, you know. We kinda went through all of that….I thought it was very appropriate. I thought it was very timely and professional. (Rose)

HR’s communication about resources and procedures helped Rose feel comfortable at work even though she was not currently experiencing unwanted attention.

As other studies have found [61], participants also described how they felt uncomfortable when they continued to have to interact with someone whom they had reported for harassment or other unwanted attention. For example, Rose described feeling anxious about having to continue to work with the person she reported:

I dreaded coming into work and having to see her and, you know, if I had an assignment with her or she even had to give me a lunch break, it affected me that way. And it kind of kept me up at night. (Rose)

Likewise, participants described feeling validated, supported, and relieved when they were given the option *not to interact* with that person anymore, whether that be through firing the person, moving them to a new location, or removing a customer. When asked how she felt when her boss told a harassing customer that he could no longer come in the store, Alexis, the barista, said:

I was elated, I was like oh my gosh, I could go wherever I want. I can bend over without a problem…It was a dream….I was so like relieved to not have to worry about him coming in, having to deal with him. (Alexis)

For Alexis, bending over without worrying about the man who had repeatedly harassed her “was a dream,” and she described feeling comfortable, instead of unsafe, at work. This comfort was due to her boss taking her report seriously and preventing the harassing customer from returning.

Importantly, participants felt upset when they themselves were penalized after trying to ensure they were separated from the person who harmed them. For example, Erica described how she felt penalized after reporting someone for harassment when her manager decided to move her (Erica) to a new facility a 30-minute drive away from her current job:

It was 30 minutes away and I was like ‘Really?’ So that was like an inconvenience for me, you know...Pretty much I had to take the transfer. She’s like ‘I [am] transferring you.’ There was no talking about it. She was just like ‘You’re being transferred.’ (Erica)

Finally, participants reported feeling supported and satisfied when organizations took action against those who had harassed them. When asked how she felt when her employer fired a colleague whom she had reported, Mai said:

I felt good when I found out. I said, ‘Yeah, I think that’s the right thing’ because, you know, if he’s doing that to me, right, later on, if we hire more female[s], then he is going to do that to other people and think that it’s fine. …Yeah, so he’s like, ‘we rather lose him than lose you’… And I said, ‘well, I appreciate that, that means a lot, you know?’ (Mai).

Mai “felt good” when her leadership openly supported her and emphasized her value to the organization using both words and action. She also believed this would help prevent harassment for future women employees.

## Discussion

This research points both to the considerable harm perpetrated by sexual harassment at work and to the organizational practices that can mitigate such harm. Like the early literature on institutional courage [53], we find that organizational practices can reduce harm from sexual harassment. In this paper, we asked: what do victimized people believe are effective organizational practices to address the problem of sexual harassment at work? We find that workers who have experienced unwanted attention at work believe organizations can mitigate harm from harassment by (1) setting clear expectations and (2) responding appropriately any time they learn of harassing behaviors within their organization. Our data suggest that people who have experienced workplace sexual harassment prefer *clear and consistent messaging* rather than effective organizational *responses* because messaging might reduce harm in the first place. Our participants described how organizations might prevent sexual harassment by making clear to workers that they are expected to treat each other with respect; effective sexual harassment education may accomplish this aim [38, 39, but see [40]]. Organizational efforts that outline behavioral expectations may prevent incidents of sexual harassment at work by establishing norms. Further, prevention should send the message that organizations *will support* workers if behavioral expectations are violated. Mai, mentioned above, described how, when “the company is up front about, like okay, you know, we never want you to feel that way, right? We’re here for you”, workers feel safe because they believe that if they experience sexual harassment at work, their employer will support them.

When up-front communication from leaders or anti-harassment education does not completely prevent harassment, participants also described how organizations can effectively respond to instances of harassment when they learn of them. First, participants desired an independent actor outside of the organizational hierarchy to whom workers can report incidents in confidence. This reporting process can help prevent abuse of power structures. Participants also described how they felt supported when their employers provided verbal acknowledgement and services in response to people who report sexual harassment at work. Several participants described how hearing an apology from the organization felt – or they imagined would feel – especially supportive. Finally, participants described how they felt supported when their employer followed up with appropriate actions that offered support to victims, separated the victim from the accused perpetrator, and reprimanded workers who harass others.

This study contributes to the literature on workplace sexual harassment. Sexual harassment can cause emotional and psychological damage to the victimized person, and workplaces suffer due to lost productivity. In addition, employers have a legal and ethical responsibility to protect their employees from harassment. The literature has identified factors that may contribute to workplace sexual harassment, such as steep hierarchies, a historic lack of diversity, and weak oversight and accountability. We expand the literature, centering the voices of people who have experienced sexual harassment at work. Our study design allowed us to consider perspectives that are diverse by gender, age, race, and job type, analyzing themes across these markers. Our respondents made no claim to understanding the complete landscape of the legal, policy, and regulatory context in which sexual harassment occurs. But by sharing their experiences of sexual harassment and their ideas for practices to help victimized people feel supported, they provide valuable information for the community of researchers and practitioners seeking to understand the conditions that may mitigate harms from sexual harassment.

This study has several limitations. First, we utilize in-depth interview data, which are useful for understanding how people make sense of their experiences but are subject to recall and social desirability biases. Our data do not provide a statistically generalizable account of sexual harassment but rather explore what organizations can do to reduce the harm from harassment from the perspectives of people who have experienced workplace harassment. Future research could explore the themes we identify here among larger groups of people. In addition, our sample was made up almost exclusively of workers in the San Francisco Bay Area. Future studies could examine whether workers who have experienced sexual harassment in other geographical areas offer similar accounts and descriptions of how organizations might reduce the harm of harassment. Most workers in our sample also worked in the service industry, limiting our understanding of the experiences of workers in other industries. Additionally, future research can better examine how people of different social identities (e.g., by race, gender, age) think about how their workplaces can prevent and respond to sexual harassment in the workplace. We hope that future research will build on this research and address these additional areas this study did not explore.

In conclusion, several of our findings are in line with and contribute to ongoing work to identify specific steps to support institutional courage [53,62,63]. Our findings show that workers believe organizations might prevent harassing behaviors through clear, consistent messaging from leadership that specifically addresses harassment and sets expectations for respectful behavior in the workplace. Participants believed this messaging, combined with verbal follow-up and appropriate disciplinary actions for the perpetrator and supportive services for the targets of harassment, may deter potential harassers by setting clear boundaries and empower workers who experience sexual harassment to bring the issue to leadership for resolution. By prioritizing the voices of workers who have experienced sexual harassment at work, we shift the focus from employer liability, where discussions of sexual harassment often center, to worker well-being and workplace equity.

## Supporting information

S1 File Interview Protocol. (DOCX)

S2 File Interview Codebook. (DOCX)
