# Supplementary material for: Sexual harassment at work: Targets’ perspectives on prevention and response
Source: PLoS One. 2026 Jul 10;21(7):e0352783. doi: 10.1371/journal.pone.0352783 (PMC13354074; doi:10.1371/journal.pone.0352783)
Supplement: S1 File — (DOCX) [file pone.0352783.s001.docx]

**S1 File. Interview Protocol**

**Find out during intro conversation:**

How did you find out about the study?

**Intro questions:**

- Tell me about your workplace. How long have you worked there?
- What are your relationships like with your colleagues?
- Walk me through a typical day: What do you do? Who do you interact with?

**SH experiences** (ask questions one at a time)

- Have you ever experienced unwanted attention at work because of your gender?
  - If you feel comfortable, can you tell me a bit about what happened?
  - What happened in the aftermath? How did you respond, if at all?
  - Did you consider reporting it to your company? What was your thought process about the pros and cons of reporting?
    - [If reported:] How did the person you reported to respond?
    - Did you tell any family members? Friends? Co-workers? Anyone else? How did they respond? Did they encourage or discourage you from reporting it to your company?
    - What was your company’s response after that?
    - Did your company acknowledge what had happened?
    - Did your company apologize for your experience?
    - Did you experience any kind of retaliation or kudos for speaking up?
    - What would have been the best way for your company to have handled the issue?
  - How did the incident affect your ability to do your work, if at all?
  - How did the incident affect your intention to stay with the company, if at all?
- Have you ever observed anyone else experiencing unwanted attention because of their gender in your workplace?
  - If you feel comfortable, can you tell me a bit about what happened?
  - What happened in the aftermath? How did you respond, if at all?
- Do people in your workplace ever make statements or comments about women or men that you find problematic or offensive?
  - [Same follow-up questions as above]
- What about transgender or gender nonbinary people?
- Do people ever have discussions about sex or make jokes about sex that you find uncomfortable?
  - [Same follow-up questions as above]
- Have you ever seen sexual images or other media about sex in your workplace that made you uncomfortable?
  - [Same follow-up questions as above]
- Have you ever experienced unwanted romantic or sexual attention at work?
  - [Same follow-up questions as above]
- Has anyone ever said or implied that if you had sex with them, you would be professionally rewarded?

**Climate level** (ask questions one at a time)

- [Does/did] your workplace have a policy for reporting gender-based treatment including sexual harassment? What do you know about that policy? What is your sense of whether the written policy matches your company’s actual practices?
- [If reporting at current workplace has not already been covered in the interview] Imagine someone at your company is experiencing sexual harassment and decides to report it. What do you think that process looks like for them?
  - How do you think that person’s report would be received and handled by the company?
- Does your workplace offer education or trainings about gender-based treatment or sexual harassment? Have you completed it? Can you tell me what you remember? How have people responded? How do you feel about it?
- Do you have a sense of how widespread unwanted gender-based treatment, including sexual harassment, is in your company? [If yes] How did you come to know that?

- - [If not already mentioned] Does your company conduct anonymous surveys that measure this?
  - [If yes] How accurate and effective do you think these surveys are? Are results released to employees?
- How are people who speak out against unwanted gender-based treatment or sexual harassment treated in your workplace?
- Have your company leaders taken a stance on the issue of unwanted gender-based treatment, including sexual harassment? What have they said? How informed do they seem?
- What is your sense of the resources your company has devoted to these issues?
  - Does your company use its platform or products in a way that helps to address unwanted gender-based treatment, including sexual harassment, in the wider world?
- [If not already described:] Has there ever been a time that you brought up a concern (that may or may not be related to the issue we’ve discussed today) at your company and you were satisfied with the outcome? Tell me about what happened.
- If you could wave a magic wand and make your company a better place to work with respect to gender-based treatment or sexual harassment, what would you change?

**Other identities**

- Sexual orientation often affects peoples’ daily experiences. Would you say any of the experiences you’ve described here have been affected by sexual orientation?
- Race and ethnicity also often affect peoples’ daily experiences. Would you say any of the experiences you’ve described here have been affected by those things?

**Wrap up**

- You’ve probably noticed from the questions I’ve asked today that our research team is interested in how organizations address issues of gender-based treatment and sexual harassment. Those were all the questions I have for you today. Do you have anything else to add?

**Demographics**

- What is your gender? _______________________
- What is your age? _______________________
- What is your racial or ethnic background? _______________________
- What is your highest level of education? _______________________
- Are you married or partnered? _______________________
- What is your sexual orientation? _______________________
- How long have you worked at your current company? _______________________
- How long have you worked in your industry? _______________________
- How many hours do you work per week? _______________________

*[If over video chat: How would you like to receive the gift card – in the mail or send a photo with the #?]*
