# Supplementary material for: Sexual harassment at work: Targets’ perspectives on prevention and response
Source: PLoS One. 2026 Jul 10;21(7):e0352783. doi: 10.1371/journal.pone.0352783 (PMC13354074; doi:10.1371/journal.pone.0352783)
Supplement: S2 File — (DOCX) [file pone.0352783.s002.docx]

**S2 File. Interview Codebook**

- Company culture
  - Culture of masculinity
  - Gender minority
  - Leadership
  - SH policy
  - SH training
  - Survey
- Harassment description
  - Physical harassment and/or sexual assault
  - Quid pro quo
  - Stalking
  - Verbal harassment
- Institutional betrayal
  - Inadequate response post investigation
    - No penalty
    - No workplace changes
  - No clear reporting pathway
  - Report dropped
    - Cursory investigation
    - Ignored or minimized
  - Workplace retaliation
- Institutional courage
  - Courageous sexual harassment policy
  - Courageous sexual harassment training
  - Moderation of impact
  - Respondent suggestions for IC
  - Serious response
    - Penalty for perpetrator
    - Thorough investigation
  - Transparency
  - Validation – apology
  - Victim relief
- Job description
  - Customer service
  - “Gig” work
  - Hourly scheduled employee
  - Salaried work
- Perpetrator characteristics
- Victim characteristics
- Victim response to harassment
  - Avoidance
  - Confrontation
  - Labelling harassment
  - Quitting
  - Reporting
    - Boss
    - HR
    - Not reporting
  - Soldiering on
